# Effects of forests, roads and mistletoe on bird diversity in monoculture rubber plantations

**DOI:** 10.1038/srep21822

**Published:** 2016-02-23

**Authors:** Rachakonda Sreekar, Guohualing Huang, Mika Yasuda, Rui-Chang Quan, Eben Goodale, Richard T. Corlett, Kyle W. Tomlinson

**Affiliations:** 1 Center for Integrative Conservation, Xishuangbanna Tropical Botanical Garden, Chinese Academy of Sciences, Menglun, Yunnan, China; 2 School of Biological Sciences, University of Adelaide, Australia; 3 Products Research Institute, Tsukuba, Ibaraki, Japan; 4 College of Forestry, Guangxi University, Nanning, Guangxi, China

## Abstract

Rising global demand for natural rubber is expanding monoculture rubber (*Hevea brasilensis*) at the expense of natural forests in the Old World tropics. Conversion of forests into rubber plantations has a devastating impact on biodiversity and we have yet to identify management strategies that can mitigate this. We determined the life-history traits that best predict bird species occurrence in rubber plantations in SW China and investigated the effects of surrounding forest cover and distance to roads on bird diversity. Mistletoes provide nectar and fruit resources in rubber so we examined mistletoe densities and the relationship with forest cover and rubber tree diameter. In rubber plantations, we recorded less than half of all bird species extant in the surrounding area. Birds with wider habitat breadths and low conservation value had a higher probability of occurrence. Species richness and diversity increased logarithmically with surrounding forest cover, but roads had little effect. Mistletoe density increased exponentially with rubber tree diameters, but was unrelated to forest cover. To maximize bird diversity in rubber-dominated landscapes it is therefore necessary to preserve as much forest as possible, construct roads through plantations and not forest, and retain some large rubber trees with mistletoes during crop rotations.

Human-driven habitat loss due to agricultural intensification is currently expanding faster than any time in the past 50 years, with a conservative estimate of c. 120 million hectares of additional land, mostly in developing nations, required to meet the projected demand by 2050^1^. Croplands particularly threaten the tropical forests that hold the majority of the world’s species^2^. The future of global biodiversity will therefore increasingly be placed in the hands of agricultural policy makers^3^. Assessments of the impacts of croplands on native ecosystems are an urgent priority for conservation, especially in tropical East Asia where both species richness and conversion of forests to monoculture plantations are among the highest globally^4^,^5^. Rubber is the most rapidly expanding tree crop in mainland SE Asia (with 84% of the global total rubber area), and an estimated 4.3–8.5 million hectares of additional rubber plantations are required to meet global demand by 2024^6^. This threat is imminent: for example, the Cambodian government has recently allocated 346,000 hectares inside 23 protected areas to rubber companies^7^.

Conversion of natural forests into monoculture rubber plantations reduces species richness and changes the composition of animal assemblages^8^,^9^,^10^,^11^,^12^,^13^, but we are yet to identify conservation strategies that could improve biodiversity within monoculture rubber landscapes. Studies in oil palm plantations have shown that nearby natural forests can enhance biodiversity in palm plantations, but not enough to maintain original assemblages^14^,^15^,^16^,^17^. However, riparian reserves within oil palm plantations were important to maintain riparian ants, dung beetles and freshwater fish communities^5^,^18^. Research is still needed to understand how neighbouring forests support biodiversity in rubber^6^.

Millions of kilometers of new roads are anticipated by 2050, partly to facilitate movements of resources in and out of these plantations^19^. However, roads often open up tropical forest regions to colonization and exploitation^20^. Roads escalate forest degradation, spread invasive species, act as a barrier for many sensitive forest species, and provide easy access for overexploitation^20^. However, roads within plantations may potentially increase animal diversity by creating bordering plant communities distinct from the monoculture plantations^21^. Research is still needed to determine if roads can have a positive or negative effect on the biodiversity in rubber.

Birds are often used as a proxy for the biodiversity value of human-dominated landscapes because they 1) are easy to survey, 2) are diverse, with better known life history attributes than any other group^22^, 3) have diet guilds that respond differently to different threats^22^,^23^,^24^,^25^,^26^, and 4) provide multiple ecosystem functions, including seed dispersal and predation, pollination, scavenging, nutrient deposition, and pest predation^22^,^23^. Though birds are the most studied vertebrate group, studies on the effects of habitat change on their life history traits are still scarce^27^,^28^ and research is needed to determine the life-history traits that best predict species occurrence in rubber. Small insects pollinate rubber trees (*Hevea brasiliensis*) and seeds are dispersed explosively, but ten families of nectarivorous and frugivorous birds were recorded in monoculture rubber in SE Asia^11^,^12^,^13^. We suspected that mistletoes, a diverse group of parasitic plants that are found on rubber trees, provide key resources in rubber plantations through provision of abundant fruits and nectar^29^.

In this paper, 1) we compiled a comprehensive checklist of resident birds that occur in the entire landscape (forests, villages, rubber and banana plantations) and compared it with a checklist of resident birds that occur in rubber plantations to determine life history traits that predict occurrence probabilities of birds in rubber plantations. 2) We conducted 52 bird point counts (repeated four times) across a distance gradient from roads and primary forests within monoculture rubber to determine the effects of forest and roads on bird diversity and composition (Fig. 1). We modeled bird richness at each point as a function of forest cover and distance to roads. It has been shown that species richness increases non-linearly with increasing forest cover or distance to roads^30^,^31^ and that thresholds can be detected in some cases^32^,^33^, so we also determined the shape of model fits and searched for thresholds of forest cover and road distance, if any. 3) Finally, we determined the effects of forest cover and rubber diameter at breast height (DBH) on mistletoe densities in rubber plantations, and made focal point observations to determine if frugivorous and nectarivorous birds feed on them.

## Results

### Trait determinants of bird occurrence

Among the 156 extant resident diurnal bird species in the Menglun landscape between 2011 and 2015, we recorded 58 species (37.1%) in rubber (Table S1). Considering forest birds only, we recorded 33.7% (33 of 98 recorded in forests) at least once in rubber (Table S1). Bird species occurrence probability increased with habitat breadth (X^2^_(1,147)_ = 10.45, *P* = 0.001; Fig. 2), but was not influenced by forest preference (X^2^_(1,147)_ = 0.09, *P* = 0.75), body size (X^2^_(1,147)_ = 0.78, *P* = 0.37) or diet type (X^2^_(4,147)_ = 3.1, *P* = 0.54).

### Environmental determinants of bird diversity

We recorded 3140 individuals of 45 resident and migrant species in rubber during point counts (Table S2). We only considered 32 species (71%; 3076 individuals) in the analysis, because we recorded the other 13 species in fewer than four points (Table S2; including the excluded species did not change results). We used original species richness instead of extrapolated richness estimators because the former had reached an asymptote (Fig. S1). Additionally, the extrapolated richness at each sample point of all species (r = 0.84, *P* < 0.001), frugivores (r = 0.98, *P* < 0.001) and insectivores (r = 0.89, *P* < 0.001) was highly correlated with original species richness.

Bird species richness and diversity in monoculture rubber plantations increased logarithmically with forest cover, as did frugivore and insectivore richness and diversity (Table 1; Fig. 3). The piecewise linear regressions estimated threshold values of forest cover as 4.01 ha (95% CIs: 1.1–6.91), 5.92 ha (95% CIs: 1.2–10.63) and 1.86 ha (95% CIs: 0.69–3.04) within the radius of 50 m from the point (78 ha) for the richness of all species, frugivores and insectivores respectively, which increased their species richness by c. 4, 3 and 3 species per point respectively. Similarly, the piecewise linear regressions estimated threshold values of forest cover as 2.76 ha (95% CIs: 0.97–4.55), 4.63 ha (95% CIs: 1.89–7.36) and 1.84 ha (95% CIs: 0.69–2.98) for the diversity of all species, frugivores and insectivores respectively (Fig. 3).

Forest cover also influenced bird species composition (*P* = 0.001; Fig. 4). Among the 32 species used in the analyses, the occurrence probability (OP) of 11 species increased with forest cover (Table 1). Very few responded to distance to roads. The OP of Grey-headed Canary Flycatcher (*Culicicapa ceylonensis*) increased with distance to the road, and the OP of Crimson Sunbird (*Aethopyga siparaja*) and Magpie Robin (*Copsychus saularis*) decreased with distance to the road (Table 1).

### Determinants of mistletoe densities

We recorded 95 mistletoe individuals after scanning 800 rubber trees in 20 different rubber plots. The number of mistletoes in a plot increased exponentially with the DBH of the trees (β ± SE = 0.034 ± 0.005, *P* < 0.001; Fig. 5), but not with forest cover (β ± SE = 0.000002 ± 0.000003, *P* = 0.456). We observed 16 species of resident frugivores and nectarivores in rubber between 2011 and 2015, of which we recorded 13 during point counts. Frugivores and nectarivores comprised 47% of total bird abundance (1439 of 3076) in rubber. We observed 68 individuals belonging to five species (four families) feeding on flowers and/or fruits of mistletoes during our mistletoe observations (Table S1).

## Discussion

Our study shows that natural forests enhance bird diversity within nearby monoculture rubber plantations. However, less than half of all the bird species in the study area were found in rubber even when forests where in close proximity. Habitat breadth was the most important trait that increased bird occurrence. The most parsimonious model reveals that the difference in occurrence probability between birds with habitat breadth one and seven was 57% (Fig. 2), suggesting that the birds that use multiple habitats, and are therefore typically of low conservation concern, tend to occur in rubber. Therefore, retention of the remaining forest patches not only improves bird diversity in the neighboring rubber plantations, but also protects the 66% (65 of 98) of forest bird species that do not use plantations. These results are in line with studies conducted in other monoculture rubber plantations, which also showed that less than half of the bird species occur in monoculture rubber^6^,^34^. However, studies conducted in jungle rubber (low intensity multi-cropping systems that contain natural colonizing vegetation) showed that such practices could retain more than half of the bird species^6^,^35^.

Forest cover alone explained 43% of the species richness variation in rubber (Table 1). The most parsimonious model reveals that an increase in forest cover within 500 m radius of a sampling point from 0.35 to 39 ha increased the average bird richness by eight species (Fig. 3). Forest cover had similar influences on frugivore and insectivore species within rubber (Fig. 3), but based on the estimated threshold values, frugivorous bird species that occur in rubber required more forest area (5.92 ha) than insectivores (1.84 ha; Fig. 3). Typically frugivores have lower fragmentation sensitivity and travel longer distances between food resources^27^, however frugivores in rubber may require higher surrounding forest cover due to the lack of adequate resources in rubber. These results are in line with previous studies, which showed that the insectivorous bird composition in rubber was more similar to primary forests than it was for frugivorous species (see Mang & Brodie^34^). This suggests that rubber plantations are better for insectivores than frugivores. In contrast, studies showed that cocoa plantations are better for frugivores than insectivores^34^, whereas in oil palm plantations both frugivores and insectivores are equally sensitive^36^.

Road proximity had little effect on most birds in rubber plantations because species that occupied them were habitat generalists (Fig. 2). Grey-headed Canary Flycatcher (*Culicicapa ceylonensis*) avoided roads, but Crimson Sunbird (*Aethopyga siparaja*) and Magpie Robin (*Copsychus saularis*) preferred them. Previous studies from around the world also show that roads and their marginal vegetation can enhance foraging and roosting opportunities, and minimize predation pressure for a few bird species, especially in unnatural anthropogenic habitats like rubber^21^. Therefore, building roads through plantations will affect biodiversity less than if they are built through forest^20^.

Maintenance of large rubber trees with mistletoes might further enhance frugivore and nectarivore diversity. Around half of the total bird abundance in rubber plantations consisted of frugivores and nectarivores, although rubber produces neither fleshy fruits nor accessible nectar. Luo *et al.*^37^ showed that the Plain Flowerpecker (*Dicaeum concolor*) and Red-whiskered Bulbul (*Pycnonotus jocosus*) were the most important dispersers of *Dendrophthoe pentandra,* the most common mistletoe in our study area. These two-bird species alone account for 37% (536 of 1439) of all frugivores and nectarivores recorded in rubber. Additionally, Scarlet-backed Flowerpeckers (*Dicaeum cruentatum*) and Japanese White-eyes (*Zosterops japonicus*) were also commonly observed feeding on mistletoes in rubber and all four species together account for 74% (1070 of 1439) of the recorded frugivores and nectarivores. Although a 40 year rotation is optimal for rubber^38^, farmers in Xishuangbanna tend to cut down their plantations at 15–30 years in order to replant with new, higher yielding, clones (G. Huang *pers. observ.*). Such short rotations limit the build-up of mistletoe populations. Obiang and Salle^39^ have shown that mistletoe parasitism on rubber trees in Africa did not affect yield, so retaining a few old trees with mistletoes while replacing the plantation with new high yielding clones could provide a low-cost benefit to biodiversity.

Although ten families of frugivorous and nectarivorous birds were recorded in monoculture rubber in tropical East Asia^10^,^11^,^12^,^13^, only four were recorded in Xishuangbanna (Table S1). Among the others, parrots (Psittaculidae) and mynas (Sturnidae) were common in rubber plantations in Malaysia and Indonesia^11^,^12^. Indeed, Wee^40^ recorded parrots feeding on fruits of *Dendrophthoe pentandra,* the most common mistletoe in our study area^37^. The absence of these birds is almost certainly due to hunting. Parrots and forest mynas used to inhabit forests around the study area (Menglun) but have been locally extirpated or exist in very small populations (<10 individuals; possibly vagrants or escaped cage birds) due to hunting and the pet trade^24^,^41^. Hunting has been reported as a serious threat in the study area since 1985^42^. Sreekar *et al.*^41^ recently showed that birds with body sizes greater than 21 cm had a 52% extirpation probability in this area, primarily due to hunting, and that hunting alone increased frugivore extirpation probability in Menglun by around two-fold^24^. Therefore, hunting might have extirpated or reduced populations of a few larger frugivore species that might have otherwise occurred in rubber plantations.

**Figure 1:**
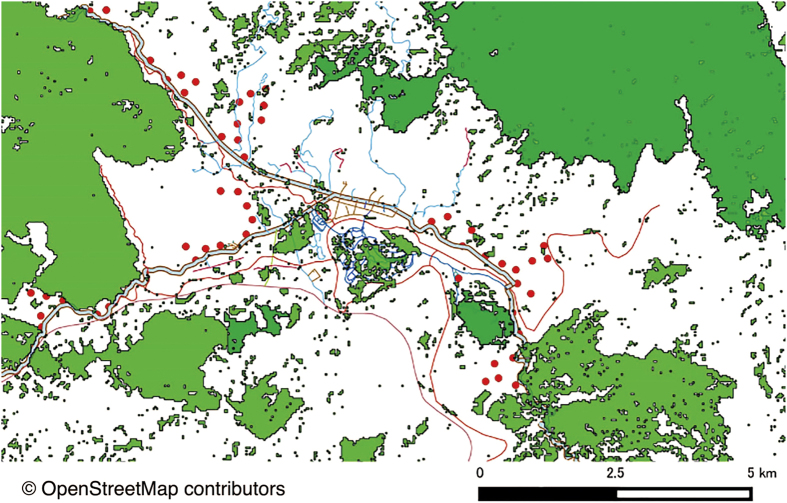
Map of Menglun landscape, Xishuangbanna Prefecture, Yunnan, China, in 2010. In the map, green is forest cover, white is rubber plantations and other human land-use, red circles are points sampled for birds in rubber, thick pale green lines are the paved roads along which sampling was conducted, thin light blue lines are unpaved roads, and maroon lines are a river and streams. We produced the forest-cover map for the study landscape by calculating the Normalized Difference Vegetation Index (NDVI) of multispectral data from Landsat-2 Multi Spectral Scanner (MSS) and Landsat-5 Thematic Mapper (TM) images (30 m spatial resolution) acquired in February 2010 using the free and open source software QGIS 1.8.0 (http://www.qgis.org) and GRASS 6.4.3 (http://grass.osgeo.org). The Landsat images were downloaded from the open database at the Earth Resources Observation and Science centre (EROS), U.S. Geological Survey (USGS; http://eros.usgs.gov), and road data from the open database OpenStreetMap (http://www.openstreetmap.org). The map tiles of road data are available under the Creative Commons Attribution-ShareAlike 2.0 licence (CC BY-SA; http://www.openstreetmap.org/copyright).

**Figure 2:**
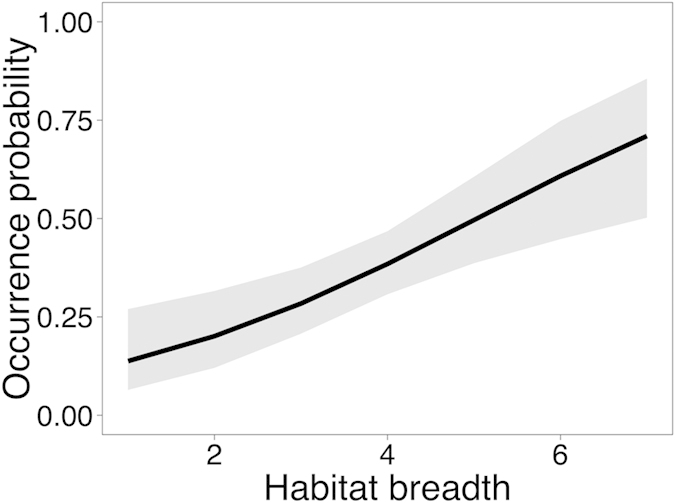
Occurrence probability of Menglun birds in monoculture rubber plantations as a function of habitat breadth. The black line is the prediction of the model fitted to the data and the shaded grey area is its 95% confidence interval.

**Figure 3:**
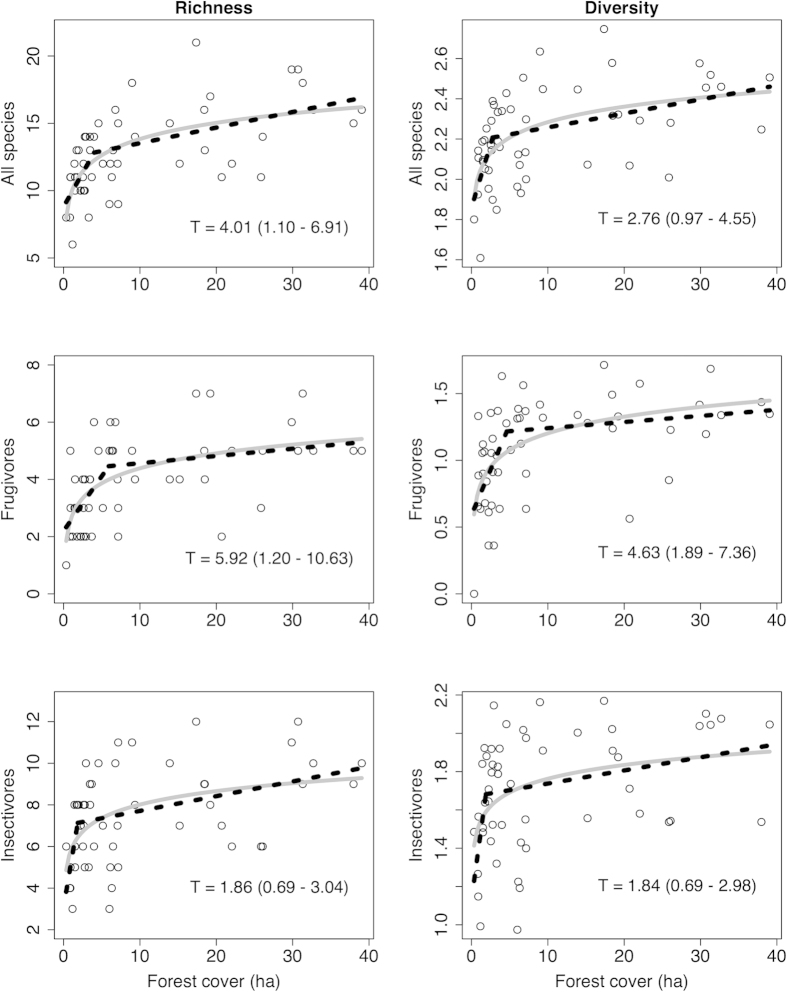
All, frugivorous and insectivorous bird richness and Shannon diversity as a function of forest cover (in hectares) within 500 m from a sampling point. The grey lines are predictions of the general linear models fitted to the data and the dashed black lines are predictions of piecewise linear regressions. The ‘T’ in each plot is the threshold value of forest cover with 95% confidence intervals in parenthesis.

**Figure 4:**
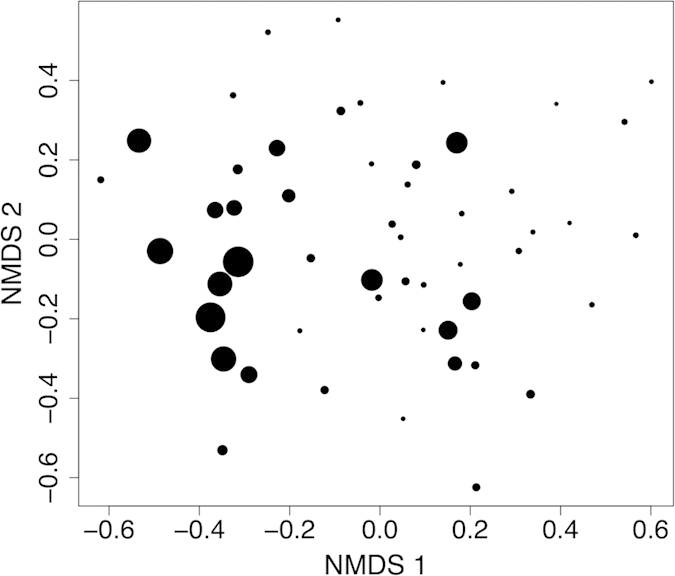
A non-metric dimensional scaling (NMDS) based on the Bray-Curtis distance with a two-dimensional solution of the bird assemblages in rubber plantations of Menglun, Xishuangbanna, Yunnan, China. The size of the circle is proportional to the forest area (in hectares) within 500 m radius of a sampling point. The ordination diagram is for visualization only. We conducted all tests using multivariate generalized linear models (see text for statistical analyses).

**Figure 5:**
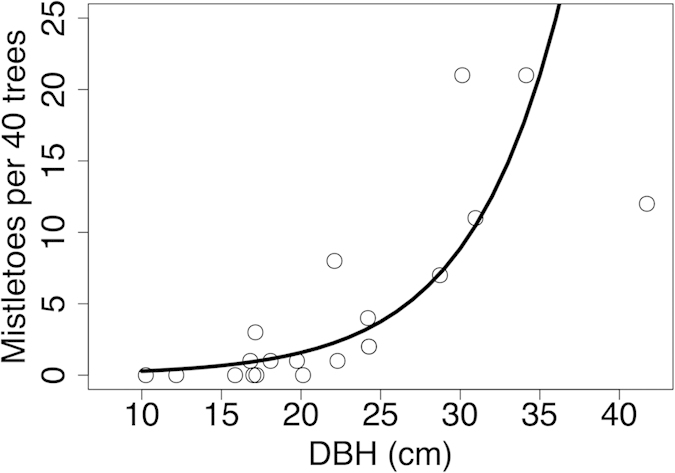
Density of mistletoes (per forty trees) in monoculture rubber plantations as a function of rubber tree diameter at breast height (DBH in cms). The black line is the prediction of the model fitted to the data.

Some limitations of this study need discussion, which also can guide future research. First, our study placed sampling points near large forest patches (>1000 ha; Fig. 1), but a question for future research is the role of degraded smaller forest fragments (<20 ha) in retaining rubber birds. Previous studies have already shown such fragments can conserve birds and trees^43^,^44^. Second, the result that a third of the bird species persist in rubber represents a best-case estimate, as we do not know if they can complete their entire life cycle there. It is possible that the individuals in rubber represent sink populations. Therefore, future studies should use spatio-temporal counts, tracking techniques and nest surveys to understand the life cycles of these species in rubber. Third, we also recommend investigating a larger range of organisms to understand how to effectively manage and conserve biodiversity in landscape mosaics with monoculture rubber plantations. Finally, we suggest conducting mistletoe exclusion experiments to better understand the importance of mistletoes in rubber for frugivorous and nectarivorous birds.

Our results suggest that natural forests are irreplaceable for biodiversity conservation as more than half of the local bird species do not occupy monoculture rubber even when in close proximity to natural forests (<100 m). The birds that occur in rubber are common species that tend to use multiple habitats and are of low conservation concern. In order to maximize biodiversity in rubber-dominated landscapes, local smallholders, rubber companies and governments should work together. First, based on the evidence summarized here, there is an obvious need to preserve as much of the remaining natural forest as possible. Second, new roads should be placed through rubber plantations where they appear to have little effect on bird diversity, and outside the remaining forests, where they are known to have negative impacts on diversity^20^. Third, farmers should be encouraged to retain at least a few large rubber trees (>25 cm DBH) with mistletoes within plantations during rotations.

## Methods

### Study area

We carried out our study in monoculture rubber plantations within 10 km radius of Xishuangbanna Tropical Botanical Garden (XTBG) at Menglun, Xishuangbanna Prefecture, Yunnan Province, tropical SW China (21°55′N, 101°15′E). In this area, a change in land tenure regulations resulted in a massive expansion of smallholder, monoculture rubber plantations that resulted in around 65% forest loss between 1976 and 2010^24^. Hunting is also common in the area and increased bird extirpations by at least 1.3 to 1.6 fold over deforestation alone^24^. Currently, four large primary forest patches (each >1000 ha) contribute >90% of remaining forest cover in the study area^24^ (Fig. 1).

### Trait determinants of bird occurrence

We compiled a list of all resident birds that occurred in the region by conducting exhaustive bird surveys in the study area (10 km radius from XTBG) between 2011 and 2015. The first author and three other experienced birders, who lived in the study area for four years, conducted all bird sampling by repeatedly walking through forests, villages and rubber plantations. We also shared observations and sightings with other amateur and professional ornithologists who visit the study area in large numbers (c. 400 birdwatchers per year). We used a mixture of methodologically constrained and unconstrained bird sampling, which is an efficient way to obtain high coverage of birds in an area^45^. We included only the resident diurnal terrestrial species in the rubber bird-list because we did not conduct systematic surveys for nocturnal and migratory species. Initial surveys did not include migratory species and our migratory bird list does not appear to be complete; therefore, we excluded them prior to analysis. Furthermore, a list of resident bird species that occurred in rubber plantations was also prepared to compare with the overall list and determine how best to explain which species occur in rubber. Our overall and rubber species accumulation curves had reached an asymptote at the end of our four-year (>2500 man-hours) bird survey of the study area (see Sreekar *et al.*^24^). Thus, we are confident that our inventories are nearly complete and the bird species that could not be found in rubber can be reasonably classified as absent.

We collected data on four life-history traits of birds using Yang *et al.*^46^ and Robson^47^ to investigate the correlates of occurrence in rubber plantations among bird species. These were forest specialization (specialist or non-specialist), body size (bill to tail length), habitat breadth (observed number of habitats the species occurs in; range: 1–8) and primary diet type (frugivore, insectivore, carnivore, granivore, nectarivore). We used a generalized linear model (GLM) with binomial error structure to model the effects of bird traits on bird occurrence probability in rubber, and an ANOVA (type III sum of squares) to determine the variance explained by each variable.

### Environmental determinants of bird diversity

We produced forest-cover maps for the study landscape by calculating the Normalized Difference Vegetation Index (NDVI) of multispectral data from Landsat-2 Multi Spectral Scanner (MSS) and Landsat-5 Thematic Mapper (TM) images (30 m spatial resolution) acquired in February 2010. We made sure that the selected images had the least cloud cover possible. Rubber trees are briefly leafless at this time, enhancing the contrast with the largely evergreen rain forest. NDVI values >0.1 were converted into 1 and everything else into 0. We layered roads upon the forest cover map using images downloaded from OpenStreetMap and ground-truthed road data (see Fig. 1). Other human altered land types in the study area include villages, river and banana plantations. We converted the resulting raster to a vector shape file as a forest-road map to calculate the distance to nearest paved roads, distance to nearest forest (>1000 ha), forest cover within 250 m, 500 m, 750 m and 1000 m from the sampling point (Table S3). Forest cover (in ha) within 500 m radius of a sampling point was highly correlated with distance to nearest forest (r = -0.77; *P* < 0.001), forest cover at 250 m (r = 0.79; *P* < 0.001), 750 m (r = 0.90; *P* < 0.001) and 1000 m (r = 0.81; *P* < 0.001), so we only used forest cover within 500 m radius (hereafter ‘forest cover’) in the model.

Using GIS and ground-truthing, we placed 52 point counts in rubber at different distances from paved road (13–1272 m) and forest patches (21–1927 m) in order to maximize the variation between these factors, and minimize their correlation (Fig. 1, Table S3). The minimum distance between each point was 300 m, we sampled each four times in the winter and spring seasons (December 2014–March 2015). All point counts were conducted between 550 and 650 m elevation in rubber plantations with little or no undergrowth. Younger plantations (<10 cm DBH) were avoided as they had relatively open canopies and were structurally different. We recorded all bird detections (sighting and aural) using ten-minute fixed radius (50 m) point counts, conducted between 8:00 and 10:00 AM (Beijing Standard Time). We used a Marantz solid-state recorder PMD661 MK-II (Marantz America, Mahwah, New Jersey, USA) with a Seinheiser microphone (Seinheiser, Wedemark, Germany) to record calls during the point count and identified unrecognized calls in the lab. We recorded both migrant and resident birds during point counts and used a species accumulation curve to assess the effectiveness of sampling effort.

To evaluate the effectiveness of sampling effort, we transformed the original species richness into estimated richness using Chao’s non-parametric estimator^48^. We used Pearson’s correlation test to determine the correlation between original and Chao’s estimated richness. We removed species that were detected fewer than four times from the data to reduce the influence of accidental occurrences (including the excluded species did not change results)^26^,^41^,^49^.

We used general linear models to determine the effects of distance to roads from the sampling point (13–1272 m) and forest cover within 500 m radius of the sampling point radiuses (0.35–39 ha) on species richness and diversity of all birds, frugivores only and insectivores only, respectively. Road distance (in meters) and forest cover (in hectares) were log_e_ transformed prior to analysis to improve their spread so that they form good linear fits with the response variable. We used the Shannon diversity index as a measure of species diversity because, unlike Simpson’s diversity index, it does not give more weight to common or dominant species^50^. We checked for spatial autocorrelation among model residuals using Moran’s I and found no evidence of autocorrelation (*P* > 0.1). To determine the effects of distance to road and forest cover on the occurrence probability (presence-absence) of individual species, we used generalized linear models with binomial error structures.

We generated full model, null model and models with all valid combinations of the explanatory variables (road distance and forest cover). There were no interaction effects between forest cover and distance to road in the models, so it was not included in the combinations of explanatory variables. We compared and ranked models using the Akaike Information Criterion (AIC_c_)^51^. We also calculated Akaike weights (wAIC), which provide relative weights for any particular model in relation to the entire model set, which varies from 0 (no support) to 1^52^ (complete support). We summed up the wAIC of all the models containing a particular covariate (covariate weight) within the subset to identify the covariates that had the strongest influence^41^. We present model-averaged estimates and their unconditional standard errors for covariates with weights (w) ≥0.75. We used McFadden’s pseudo-R^2^ to calculate the deviance explained by the generalized linear models^53^. After determining the best fitting covariates (w ≥ 0.75) for species richness and diversity, we additionally fitted piecewise models to determine the existence and estimates of thresholds across the chosen environmental predictors^54^.

We used a multivariate generalized linear model with negative-binomial error structure to determine the effects of distance to road and forest cover on bird community composition in rubber^55^. We assessed the significance of explanatory variables using 999 permutations of a Monte-Carlo test. We used non-metric multi-dimensional scaling based on the Bray-Curtis distance with a two-dimensional solution only to visualize changes in bird composition across rubber sites.

### Determinants of mistletoe densities

The rubber trees in Xishuangbanna briefly shed their leaves in February and produce new leaves and flowers in March^56^. We surveyed mistletoes, which were evergreen and thus conspicuous, on leafless rubber trees in a subset of 20 plots during a c. 15-day period in February 2015. We scanned forty trees around the sampling point for mistletoes and measured the diameter at breast height (DBH) for 10 rubber trees around the sampling point. At the same time, we also recorded birds we saw eating mistletoe fruits or visiting flowers. We used a GLM with negative binomial error structure to model the effects of rubber DBH and forest cover on mistletoe density in rubber.

All analyses were conducted in the programming and statistical language R 3.1.1^57^. The csv files and R codes are available on request from the corresponding author.

## Additional Information

**How to cite this article**: Sreekar, R. *et al.* Effects of forests, roads and mistletoe on bird diversity in monoculture rubber plantations. *Sci. Rep.* **6**, 21822; doi: 10.1038/srep21822 (2016).

## Supplementary Material

Supplementary Information

## Figures and Tables

**Table 1:** Model-averaged estimates and covariate weights of environmental variables (forest cover (in hectares) =forest cover within 500 m radius from the sampling points; road distance (in meters) =closest distance to the nearest non-rubber road) for bird species richness, Shannon diversity and individual species occurrence probability in Menglun, Xishuangbanna, Yunnan, China.

Response	Covariate	Model averaged estimate ± SE	Covariate weight	Deviance explained
***Species richness***				
All species	Forest cover	1.75** ± **0.29	1.00	0.43
Frugivore	Forest cover	0.74** **±** **0.15	1.00	0.35
Insectivore	Forest cover	1.02** **±** **0.26	1.00	0.23
***Shannon diversity***					
All species	Forest cover	0.11** **±** **0.02	1.00	0.32	
Frugivore	Forest cover	0.18** **±** **0.04	1.00	0.33	
Insectivore	Forest cover	0.12** **±** **0.04	0.98	0.16	
***Occurrence probability***					
Ashy Drongo	Forest cover	0.78** **±** **0.33	0.90	0.12	
Black-crested Bulbul	Forest cover	1.86** **±** **0.76	1.00	0.23	
Blue-throated Barbet	Forest cover	0.77** **±** **0.30	0.93	0.13	
Brown-cheeked Fulvetta	Forest cover	0.86** **±** **0.38	0.88	0.13	
Crimson Sunbird	Road distance	−0.65** **±** **0.27	0.89	0.09	
Plaintive Cuckoo	Forest cover	1.94** **±** **0.67	1.00	0.38	
Yellow-vented Flowerpecker	Forest cover	0.91** **±** **0.49	0.75	0.13	
Great Barbet	Forest cover	1.32** **±** **0.59	0.94	0.21	
Grey-headed Canary Flycatcher	Road distance	0.59** **±** **0.28	0.82	0.07	
Indian Cuckoo	Forest Cover	0.92** **±** **0.39	0.90	0.17	
Magpie Robin	Road distance	−0.68** **±** **0.30	0.85	0.11	
Puff-throated Babbler	Forest cover	0.56** **±** **0.27	0.79	0.07	
Red-whiskered Bulbul	Forest cover	1.50** **±** **0.46	1.00	0.30	
Rufous-capped Babbler	Forest cover	1.25** **±** **0.55	0.94	0.07	

Forest cover and road distance were loge transformed prior to analysis, see methods.
