# Supplementary material for: Effects of forests, roads and mistletoe on bird diversity in monoculture rubber plantations
Source: Sci Rep. 2016 Feb 23;6:21822. doi: 10.1038/srep21822 (PMC4763173; doi:10.1038/srep21822)
Supplement: Supplementary Information [file srep21822-s1.doc]

**Effects of forests, roads and mistletoe on bird diversity in monoculture rubber plantations**

**Rachakonda Sreekar, Guohualing Huang, Mika Yasuda, Rui-Chang Quan, Eben Goodale, Richard T. Corlett, Kyle W. Tomlinson**

**Supplemental Information**

Figure S1…………………………………………………………………………………….Page 1

Table S1…………………………………………………………………..………………..Pages 2-6

Table S2……………………………………………………………………………………Pages 7-8

Table S3……………………………………………………………………………………Pages 9-10

**Fig S1.** Species accumulation curve of birds recorded in 52 plots in rubber. Each plot was re-sampled four times. The black line is randomized original bird species richness and the shaded grey area is its 95% confidence interval.

**Table S1.** Summary information of species recorded in Menglun landscape, including their taxonomic information, occurrence in monoculture rubber plantations, and their ecological traits. Rubber occurrence (1 = recorded in rubber, 0 = not recorded in rubber; checklist data collected between 2011 and 2015); Forest habitat (P=prefer forest habitat, NP=no preference); Primary diet type (Inver = invertebrates, Carni = vertebrates, Frug = fruit, Nec = nectar, Gran = seed); Body size (centimeters). We collected data on four life-history traits of birds using Yang *et al*.^34^ and Robson^35^. Taxonomy and nomenclature follow Clement’s checklist (ver. 6.9; updated in Aug 2014; http://www.birds.cornell.edu/clementschecklist/download/).

| **English Name** | **Scientific Name** | **Rubber occurrence** | **Forest habitat** | **Habitat breadth** | **Primary diet type** | **Body size** |
| --- | --- | --- | --- | --- | --- | --- |
| Ashy Bulbul | *Hemixos flavala* | 0 | P | 3 | Frug | 20.5 |
| Ashy Wood Swallow | *Artamus fuscus* | 0 | NP | 5 | Inver | 17 |
| Ashy-headed Green-Pigeon | *Treron phayrei** | 0 | P | 4 | Frug | 26 |
| Asian Palm Swift | *Cypsiurus parvus* | 1 | NP | 5 | Inver | 11.5 |
| Banded Bay Cuckoo | *Cacomantis sonneratii* | 0 | P | 4 | Inver | 23.5 |
| Bar-winged Flycatcher-shrike | *Hemipus picatus* | 0 | P | 3 | Inver | 13.5 |
| Barn Swallow | *Hirundo rustica* | 1 | NP | 8 | Inver | 15 |
| Baya Weaver | *Ploceus philippinus* | 0 | NP | 4 | Gran | 15 |
| Besra | *Accipiter virgatus* | 0 | P | 3 | Carni | 29.5 |
| Black Baza | *Aviceda leuphotes* | 0 | P | 3 | Inver | 32.5 |
| Black Bulbul | *Hypsipetes leucocephalus* | 1 | P | 3 | Frug | 25 |
| Black-backed Forktail | *Enicurus leschenaulti* | 0 | P | 3 | Inver | 28 |
| Black-breasted Thrush | *Turdus dissimilis* | 1 | P | 3 | Inver | 23 |
| Black-crested Bulbul | *Pycnonotus flaviventris* | 1 | P | 3 | Frug | 19 |
| Black-headed Bulbul | *Pycnonotus atriceps* | 0 | P | 2 | Frug | 18 |
| Black-headed Sibia | *Heterophasia melanoleuca* | 0 | P | 2 | Inver | 22.5 |
| Black-naped Monarch | *Hypothymis azurea* | 0 | P | 6 | Inver | 17 |
| Black-naped Oriole | *Oriolus chinensis* | 0 | P | 5 | Inver | 25.5 |
| Black-shouldered Kite | *Elanus caeruleus* | 0 | NP | 4 | Carni | 33.5 |
| Black-throated Laughingthrush | *Ianthocincla chinensis* | 0 | P | 4 | Frug | 28 |
| Black-winged Cuckoo-Shrike | *Lalage melaschistos* | 0 | P | 4 | Inver | 23.5 |
| Blue Rock Thrush | *Monticola solitarius* | 0 | NP | 4 | Inver | 22 |
| Blue Whistling Thrush | *Myophonus caeruleus* | 0 | P | 4 | Inver | 33 |
| Blue-bearded Bee-eater | *Nyctyornis athertoni* | 0 | P | 4 | Inver | 35 |
| Blue-naped Pitta | *Pitta nipalensis* | 1 | P | 3 | Inver | 24 |
| Blue-throated Barbet | *Psilopogon asiaticus* | 1 | P | 4 | Frug | 23 |
| Blue-winged Leafbird | *Chloropsis cochinchinensis* | 0 | P | 4 | Inver | 17.5 |
| Blyth's Kingfisher | *Alcedo hercules* | 0 | P | 2 | Carni | 22.5 |
| Bronzed Drongo | *Dicrurus aeneus* | 1 | P | 4 | Inver | 22.5 |
| Brown-cheeked Fulvetta | *Alcippe poioicephala* | 1 | P | 3 | Inver | 16 |
| Buff-breasted Babbler | *Pellorneum tickelli* | 1 | NP | 5 | Inver | 17 |
| Burmese Shrike | *Lanius collurioides* | 0 | NP | 3 | Inver | 20 |
| Chestnut-crowned Warbler | *Seicercus castaniceps* | 0 | P | 2 | Inver | 10 |
| Chestnut-headed Tesia | *Tesia castaneocoronata* | 0 | P | 2 | Inver | 9 |
| Chestnut-tailed Starling | *Sturnia malabarica* | 0 | NP | 4 | Inver | 19.5 |
| Chinese Hwamei | *Garrulax canorus* | 0 | NP | 5 | Inver | 22.5 |
| Common Iora | *Aegithina tiphia* | 1 | NP | 6 | Inver | 13 |
| Common Myna | *Acridotheres tristis* | 0 | NP | 7 | Inver | 26 |
| Common Tailorbird | *Orthotomus sutorius* | 1 | NP | 6 | Inver | 12 |
| Coppersmith Barbet | *Psilopogon haemacephalus* | 1 | P | 5 | Frug | 17 |
| Crested Goshawk | *Accipiter trivirgatus* | 0 | P | 3 | Carni | 43 |
| Crested Serpent Eagle | *Spilornis cheela* | 0 | P | 3 | Carni | 65 |
| Crested Treeswift | *Hemiprocne coronata* | 0 | P | 3 | Inver | 22 |
| Crimson Sunbird | *Aethopyga siparaja* | 1 | NP | 4 | Nec | 12 |
| Dark-necked Tailorbird | *Orthotomus atrogularis* | 1 | P | 5 | Inver | 11.5 |
| Emerald Cuckoo | *Chrysococcyx maculatus* | 0 | P | 5 | Inver | 17 |
| Emerald Dove | *Chalcophaps indica* | 0 | P | 3 | Frug | 25 |
| Eurasian Blackbird | *Turdus merula* | 1 | NP | 4 | Inver | 28.5 |
| Eurasian Tree Sparrow | *Passer montanus* | 0 | NP | 4 | Gran | 14 |
| Fire-breasted Flowerpecker | *Dicaeum ignipectus* | 0 | P | 2 | Frug | 8.5 |
| Flavescent Bulbul | *Pycnonotus flavescens* | 0 | NP | 4 | Frug | 21.5 |
| Franklin's Prinia | *Prinia hodgsonii* | 1 | NP | 5 | Inver | 11 |
| Fujian Niltava | *Niltava davidi* | 1 | P | 3 | Inver | 18 |
| Gould's Sunbird | *Aethopyga gouldiae* | 0 | P | 2 | Nec | 14 |
| Great Barbet | *Psilopogon virens* | 1 | P | 3 | Frug | 32.5 |
| Great Iora | *Aegithina lafresnayei* | 1 | P | 2 | Inver | 16 |
| Greater Coucal | *Centropus sinensis* | 1 | NP | 5 | Inver | 50 |
| Green Bee-eater | *Merops orientalis* | 0 | NP | 7 | Inver | 19.5 |
| Green-billed Malkoha | *Phaenicophaeus tristis* | 0 | P | 4 | Inver | 56 |
| Grey Bushchat | *Saxicola ferreus* | 0 | NP | 8 | Inver | 14 |
| Grey Peacock-Pheasant | *Polyplectron bicalcaratum* | 0 | P | 2 | Gran | 66 |
| Grey-backed Shrike | *Lanius tephronotus* | 0 | NP | 4 | Inver | 24 |
| Grey-cheecked Warbler | *Seicercus poliogenys* | 0 | P | 2 | Inver | 9.5 |
| Grey-chinned Minivet | *Pericrocotus solaris* | 0 | P | 4 | Inver | 18 |
| Grey-eyed Bulbul | *Iole propinqua* | 0 | P | 3 | Frug | 18 |
| Grey-headed Canary Flycatcher | *Culicicapa ceylonensis* | 1 | P | 5 | Inver | 12.5 |
| Grey-headed Woodpecker | *Picus canus* | 1 | NP | 4 | Inver | 32 |
| Grey-throated Martin | *Riparia chinensis* | 0 | NP | 2 | Inver | 11.5 |
| Grey-throated Sunbird | *Anthreptes malacensis** | 0 | NP | 5 | Nec | 14 |
| Hill-blue Flycatcher | *Cyornis banyumas* | 1 | P | 3 | Inver | 15 |
| Himalayan Swiftlet | *Collocalia brevirostris* | 0 | NP | 4 | Inver | 13.5 |
| Hoopoe | *Upupa epops* | 0 | NP | 3 | Inver | 30 |
| House Sparrow | *Passer domesticus* | 0 | NP | 4 | Gran | 14 |
| Indian Roller | *Coracias benghalensis* | 0 | NP | 5 | Inver | 32.5 |
| Japanese tit | *Parus minor* | 1 | NP | 7 | Inver | 14 |
| Japanese White-eye | *Zosterops japonicus* | 1 | P | 4 | Frug | 11 |
| Large Niltava | *Niltava grandis* | 0 | P | 2 | Inver | 20.5 |
| Large Woodshrike | *Tephrodornis gularis* | 0 | P | 5 | Inver | 18.5 |
| Lesser Coucal | *Centropus bengalensis* | 1 | NP | 5 | Inver | 38 |
| Lesser Shortwing | *Brachypteryx leucophrys* | 0 | P | 2 | Inver | 12 |
| Lime-stone Wren-Babbler | *Turdinus crispifrons* | 0 | P | 1 | Inver | 19 |
| Little Pied Flycatcher | *Ficedula westermanni* | 0 | P | 3 | Inver | 12 |
| Little Spiderhunter | *Arachnothera longirostra* | 1 | P | 4 | Nec | 16 |
| Little Swift | *Apus affinis* | 1 | NP | 2 | Inver | 14.5 |
| Long-tailed Broadbill | *Psarisomus dalhousiae* | 0 | P | 2 | Inver | 25.5 |
| Long-tailed Minivet | *Pericrocotus ethologus* | 0 | P | 3 | Inver | 19 |
| Long-tailed Shrike | *Lanius schach* | 1 | NP | 3 | Inver | 26 |
| Magpie Robin | *Copsychus saularis* | 1 | NP | 7 | Inver | 20 |
| Mountain Bulbul | *Ixos mcclellandii* | 0 | P | 2 | Frug | 22.5 |
| Olive-backed Pipit | *Anthus hodgsoni* | 1 | NP | 4 | Inver | 16.5 |
| Olive-backed Sunbird | *Cinnyris jugularis* | 0 | NP | 6 | Nec | 11.5 |
| Orange-bellied Leafbird | *Chloropsis hardwickii* | 0 | P | 3 | Inver | 19.5 |
| Orange-breasted Trogon | *Harpactes oreskios* | 0 | P | 2 | Inver | 29 |
| Orange-headed Thrush | *Zoothera citrina* | 1 | P | 4 | Inver | 22 |
| Oriental Dwarf Kingfisher | *Ceyx erithaca* | 0 | P | 2 | Carni | 22.5 |
| Oriental Turtle Dove | *Streptopelia orientalis* | 0 | NP | 3 | Gran | 32 |
| Oriental White-eye | *Zosterops palpebrosus* | 1 | P | 5 | Frug | 10.5 |
| Oriental Yellow-eyed Babbler | *Chrysomma sinense* | 0 | NP | 3 | Inver | 18.5 |
| Paddyfield Pipit | *Anthus rufulus* | 0 | NP | 5 | Inver | 15.5 |
| Pied Falconet | *Microhierax melanoleucos** | 0 | P | 3 | Inver | 19.5 |
| Plain Flowerpecker | *Dicaeum concolor* | 1 | P | 4 | Nec | 8 |
| Plain Prinia | *Prinia inornata* | 1 | NP | 6 | Inver | 14.5 |
| Plumbeous Water Redstart | *Phoenicurus fuliginosus* | 0 | NP | 5 | Inver | 15 |
| Puff-throated Babbler | *Pellorneum ruficeps* | 1 | P | 4 | Inver | 17 |
| Puff-throated Bulbul | *Alophoixus pallidus* | 1 | P | 2 | Frug | 22 |
| Purple Sunbird | *Cinnyris asiaticus** | 0 | NP | 6 | Nec | 11 |
| Pygmy Cupwing | *Pnoepyga pusilla* | 0 | P | 2 | Inver | 8.5 |
| Red Collared Dove | *Streptopelia tranquebarica* | 0 | NP | 4 | Gran | 24 |
| Red Junglefowl | *Gallus gallus* | 0 | P | 3 | Gran | 71 |
| Red-billed Blue Magpie | *Urocissa erythrorhyncha* | 1 | P | 5 | Inver | 66.5 |
| Red-billed Scimitar Babbler | *Pomatorhinus ochraceiceps* | 0 | P | 1 | Inver | 23 |
| Red-whiskered Bulbul | *Pycnonotus jocosus* | 1 | NP | 5 | Frug | 19.5 |
| Rosy Minivet | *Pericrocotus roseus* | 0 | P | 3 | Inver | 18.7 |
| Ruby-cheeked Sunbird | *Chalcoparia singalensis* | 1 | P | 6 | Nec | 10.5 |
| Rufescent Prinia | *Prinia rufescens* | 1 | P | 4 | Inver | 11.5 |
| Rufous Woodpecker | *Celeus brachyurus* | 0 | NP | 4 | Inver | 25 |
| Rufous-capped Babbler | *Stachyris ruficeps* | 1 | P | 3 | Inver | 12.5 |
| Rufous-winged Fulvetta | *Schoeniparus castaneceps* | 0 | P | 2 | Inver | 11 |
| Rusty-capped Fulvetta | *Schoeniparus dubius* | 0 | P | 4 | Inver | 14.5 |
| Scaly-breasted Munia | *Lonchura punctulata* | 1 | NP | 4 | Gran | 12 |
| Scarlet Minivet | *Pericrocotus flammeus* | 0 | P | 4 | Inver | 20 |
| Scarlet-backed Flowerpecker | *Dicaeum cruentatum* | 1 | P | 5 | Frug | 8.5 |
| Silver Pheasant | *Lophura nycthemera* | 0 | P | 2 | Gran | 104 |
| Silver-breasted Broadbill | *Serilophus lunatus* | 0 | P | 2 | Inver | 16.5 |
| Slaty-backed Forktail | *Enicurus schistaceus* | 0 | P | 3 | Inver | 23.5 |
| Small Blue Kingfisher | *Alcedo atthis* | 0 | NP | 5 | Carni | 17 |
| Small Niltava | *Niltava macgrigoriae* | 0 | P | 2 | Inver | 13.5 |
| Snowy-browed Flycatcher | *Ficedula hyperythra* | 0 | P | 2 | Inver | 12 |
| Sooty-headed Bulbul | *Pycnonotus aurigaster* | 1 | NP | 5 | Frug | 20 |
| Speckled Piculet | *Picumnus innominatus* | 1 | P | 4 | Inver | 10 |
| Speckled Wood Pigeon | *Columba hodgsonii** | 0 | P | 2 | Frug | 38 |
| Spot-necked Babbler | *Stachyris striolata* | 0 | P | 3 | Frug | 16 |
| Spot-throated Babbler | *Pellorneum albiventre* | 0 | NP | 5 | Inver | 14 |
| Spotted Dove | *Streptopelia chinensis* | 1 | NP | 5 | Gran | 30.5 |
| Sreak-breasted Scimitar Babbler | *Pomatorhinus ruficollis* | 0 | P | 3 | Inver | 18 |
| Streaked Spiderhunter | *Arachnothera magna* | 0 | P | 3 | Nec | 19 |
| Striated Swallow | *Hirundo striolata* | 1 | NP | 5 | Inver | 18.5 |
| Striped Tit Babbler | *Mixornis gularis* | 1 | P | 4 | Inver | 13 |
| Thick-billed Flowerpecker | *Dicaeum agile* | 0 | P | 4 | Nec | 10 |
| Violet Cuckoo | *Chrysococcyx xanthorhynchus* | 0 | P | 5 | Inver | 17 |
| Vulvet-fronted Nuthatch | *Sitta frontalis* | 0 | P | 4 | Inver | 13 |
| White-bellied Epornis | *Erpornis zantholeuca* | 0 | P | 2 | Inver | 13 |
| White-browed Piculet | *Sasia ochracea* | 0 | P | 5 | Inver | 9 |
| White-rumped Munia | *Lonchura striata* | 1 | NP | 4 | Gran | 11 |
| White-rumped Shama | *Copsychus malabaricus* | 1 | NP | 4 | Inver | 25 |
| White-tailed Flycatcher | *Cyornis concretus* | 1 | P | 3 | Inver | 19 |
| White-tailed Robin | *Cinclidium leucurum* | 0 | P | 2 | Inver | 18.5 |
| White-tailed Warbler | *Phylloscopus davisoni* | 1 | P | 3 | Inver | 11 |
| White-throated Fantail | *Rhipidura albicollis* | 1 | P | 3 | Inver | 19 |
| White-throated Kingfisher | *Halcyon smyrnensis* | 1 | NP | 4 | Carni | 28.5 |
| Yellow-bellied Fantail | *Chelidorhynx hypoxantha* | 0 | NP | 2 | Inver | 12 |
| Yellow-bellied Prinia | *Prinia flaviventris* | 0 | NP | 4 | Inver | 13.5 |
| Yellow-bellied Warbler | *Abroscopus superciliaris* | 1 | P | 4 | Inver | 10.5 |
| Yellow-legged Button Quail | *Turnix tanki* | 0 | NP | 5 | Gran | 17 |
| Yellow-vented Flowerpecker | *Dicaeum chrysorrheum* | 1 | P | 5 | Nec | 10 |
| Yellowish-bellied Bush Warbler | *Horornis acanthizoides* | 0 | NP | 2 | Inver | 11.5 |
| Yunnan Fulvetta | *Alcippe fratercula* | 0 | P | 3 | Frug | 14 |

**Table S2.** Species recorded during point counts in monoculture rubber plantations of Menglun, Xishuangbanna, Yunnan, China. * indicates species that were recorded in less than four plots. ^a^ indicates species that were observed on mistletoes. The csv files and R-codes are available on request from the corresponding author.

| **English name** | **Scientific name** | **Abundance** |
| --- | --- | --- |
| Ashy Drongo | *Dicrurus macrocercus* | 20 |
| Asian Koel* | *Eudynamys scolopacea** | 1 |
| Barred Owlet | *Glaucidium cuculoides* | 37 |
| Black Bulbul* | *Hypsipetes leucocephalus** | 4 |
| Black-crested Bulbul | *Pycnonotus flaviventris* | 63 |
| Blue-throated barbet | *Psilopogon asiaticus* | 77 |
| Brown-cheecked Fulvetta | *Alcippe poioicephala* | 69 |
| Buff-breasted Babbler* | *Pellorneum tickelli** | 2 |
| Common Tailorbird | *Orthotomus sutorius* | 329 |
| Coppersmith Barbet | *Psilopogon haemacephalus* | 16 |
| Crimson Sunbird^a^ | *Aethopyga siparaja* | 41 |
| Dark-necked tailorbird | *Orthotomus atrogularis* | 81 |
| Dusky Warbler* | *Phylloscopus fuscatus** | 3 |
| Franklin's Prinia* | *Prinia hodgsonii** | 7 |
| Great Barbet | *Psilopogon virens* | 12 |
| Great Iora | *Aegithina lafresnayei* | 7 |
| Greater Coucal | *Centropus sinensis* | 66 |
| Greenish Warbler | *Phylloscopus trochiloides* | 9 |
| Grey-headed Canary Flycatcher | *Culicicapa ceylonensis* | 55 |
| Grey-headed Woodpecker* | *Picus canus** | 2 |
| Hill Blue Flycatcher | *Cyornis banyumas* | 103 |
| Indian Cuckoo | *Cuculus micropterus* | 15 |
| Japanese Tit* | *Parus minor** | 3 |
| Japanese Whiteeye^a^ | *Zosterops japonicus* | 378 |
| Little Spiderhunter* | *Arachnothera longirostra** | 1 |
| Long-tailed Shrike* | *Lanius schach** | 4 |
| Magpie Robin | *Copsychus saularis* | 49 |
| Olive-backed Pippit | *Anthus hodgsoni* | 17 |
| Pin-striped Tit-Babbler | *Mixornis gularis* | 185 |
| Plain Flowerpecker^a^ | *Dicaeum concolor* | 238 |
| Plaintive Cuckoo | *Cacomantis merulinus* | 13 |
| Puff-throated babbler | *Pellorneum ruficeps* | 77 |
| Puff-throated Bulbul * | *Alophoixus pallidus** | 7 |
| Red-billed Blue Magpie | *Urocissa erythrorhyncha* | 37 |
| Red-whiskered Bulbul | *Pycnonotus jocosus* | 298 |
| Rufescent Prinia* | *Prinia rufescens** | 2 |
| Rufous-capped Babbler | *Stachyris ruficeps* | 15 |
| Scarlet-backed Flowerpecker^a^ | *Dicaeum cruentatum* | 156 |
| Sooty-headed Bulbul^a^ | *Pycnonotus aurigaster* | 146 |
| Speckled Piculet* | *Picumnus innominatus** | 2 |
| White-rumped Shama | *Copsychus malabaricus* | 11 |
| White-throated Fantail | *Rhipidura albicollis* | 15 |
| Yellow-bellied Warbler* | *Abroscopus superciliaris** | 4 |
| Yellow-browed Warbler | *Phylloscopus inornatus* | 427 |
| Yellow-vented Flowerpecker | *Dicaeum chrysorrheum* | 14 |

**Table S3.** Table showing forest cover (in ha) around 500 m radius, distance (in m) to the nearest large forest patch (>1000 ha) and distance (in m) to paved road of each point.

| **PLOT** | **Forest area (500 m radius)** | **Distance to forest** | **Distance to road** |
| --- | --- | --- | --- |
| 1 | 9.3516994 | 288.3 | 374.3 |
| 2 | 31.3160584 | 109.8 | 103.8 |
| 3 | 7.1510218 | 143.8 | 632.5 |
| 4 | 6.0057537 | 300.6 | 402.8 |
| 5 | 19.2099218 | 171.8 | 46.8 |
| 6 | 25.8581743 | 98.2 | 32.5 |
| 7 | 22.0418606 | 65 | 17.4 |
| 8 | 6.475834 | 86.7 | 542.4 |
| 9 | 0.8848561 | 444.2 | 65 |
| 10 | 0.9119846 | 593.2 | 198 |
| 11 | 2.7747249 | 477.4 | 141 |
| 12 | 6.1245761 | 439.9 | 77.1 |
| 13 | 1.17 | 498.6 | 364.6 |
| 14 | 1.969729 | 1190 | 769.4 |
| 15 | 2.7719403 | 1240.2 | 878.3 |
| 16 | 1.7004939 | 894.3 | 527.1 |
| 17 | 3.4745214 | 1228 | 325.9 |
| 18 | 2.9257343 | 1204 | 85.3 |
| 19 | 6.7799804 | 1016.8 | 330.9 |
| 20 | 6.3119917 | 674.7 | 13 |
| 21 | 3.6863203 | 1115.2 | 80 |
| 22 | 1.4996473 | 965.1 | 260 |
| 23 | 2.637352 | 994.1 | 471.1 |
| 24 | 8.9678023 | 1017.1 | 742.4 |
| 25 | 1.4222106 | 1120.4 | 825.1 |
| 26 | 2.61 | 715 | 1052.6 |
| 27 | 2.2519645 | 473.4 | 1271.8 |
| 28 | 5.1409775 | 757.5 | 1001.8 |
| 29 | 2.2348101 | 666.6 | 96.9 |
| 30 | 13.9069654 | 150 | 130.2 |
| 31 | 4.5708987 | 438.6 | 413.2 |
| 32 | 3.4483703 | 336 | 277.4 |
| 33 | 18.4221883 | 151 | 37.4 |
| 34 | 17.3604067 | 199.5 | 81.2 |
| 35 | 29.8738985 | 63.4 | 68.5 |
| 36 | 18.4930259 | 197.2 | 205.3 |
| 37 | 3.9809977 | 1927.3 | 87.3 |
| 38 | 7.0680512 | 1822.3 | 282 |
| 39 | 7.1465049 | 1572.7 | 553 |
| 40 | 3.2909232 | 1272.2 | 516 |
| 41 | 0.8336166 | 1019.4 | 664.1 |
| 42 | 1.5019565 | 1232.7 | 105.4 |
| 43 | 2.5340525 | 1525.9 | 112.7 |
| 44 | 1.7792033 | 1061.1 | 29.2 |
| 45 | 0.3434021 | 880.5 | 341.8 |
| 46 | 37.971131 | 105.9 | 552.3 |
| 47 | 32.7014498 | 21.6 | 304.9 |
| 48 | 15.1999951 | 204.1 | 138 |
| 49 | 20.7062074 | 54.6 | 40.8 |
| 50 | 30.7083214 | 61.937 | 20 |
| 51 | 26.0742854 | 51.3 | 33.2 |
| 52 | 39.051298 | 37 | 87 |
